# Impaired cardioprotective function of transplantation of mesenchymal stem cells from patients with diabetes mellitus to rats with experimentally induced myocardial infarction

**DOI:** 10.1186/1475-2840-12-40

**Published:** 2013-03-03

**Authors:** Yu Liu, Zhi Li, Tao Liu, Xiaodong Xue, Hui Jiang, Jianhua Huang, Huishan Wang

**Affiliations:** 1 Department of Cardiovascular Surgery, Xijing Hospital, Fourth Military Medical University, Xian, PR China; 2 Department of Cardiovascular Surgery, Shenyang Northern Hospital, 83 Wenhua Rd, Shenhe District, 110016, Shenyang, Liaoning, China; 3 Department of Cardiothoracic Surgery, Ningxia People’s Hospital, Yinchuan, PR China

**Keywords:** Mesenchymal stem cells, Coronary artery disease, Diabetes mellitus, Myocardial infarction, Bcl-2

## Abstract

**Background:**

Diabetes mellitus (DM) exacerbates coronary artery disease (CAD) morbidity and mortality. Mesenchymal stem cells (MSCs) play an important therapeutic role in myocardial ischemic injury. However, little is known about changes in the cardioprotective characteristics of MSCs from patients with DM.

**Methods:**

Sternal bone marrow aspirates were taken at the time of coronary artery bypass graft surgery. The morphology and growth characteristics of hMSCs were observed in passage 3. Differences in gene expression profiling were measured by Affymetrix GeneChipHuman Genome U133 Plus 2.0 Arrays. Forty two adult male rats with experimentally CAD were randomized into three groups. MSCs from patients with CAD+DM or CAD were injected into the infarcted myocardium. Control animals received culture medium. Echocardiography, TUNEL, immunohistochemistry and Western-blot analysis were performed 4 weeks after transplantation.

**Results:**

Growth curves showed that proliferation of hMSCs in the CAD+DM group was significantly lower than in the CAD group. Nine transcripts of genes related to apoptosis containing Bcl-2 were found to differentiate the two groups. Transplantation of hMSCs in the infarcted border zone improved cardiac function, but DM partly impaired this effect. Similar results were observed from TUNEL, immunohistochemistry and Western-blot analysis.

**Conclusions:**

hMSCs from patients with CAD+DM and CAD alone both have proliferative properties. Transplantation of hMSCs ameliorate heart function, but proliferative ability and myocardial protection decrease significantly in MSCs obtained from patients with CAD+DM compared with cultures from patients with CAD alone, possibly as a result of differences in Bcl-2 protein expression and reduced anti-apoptosis.

## Background

Myocardial infarction (MI) and diabetes mellitus (DM) are serious diseases with high morbidity and mortality. It was estimated in 2010 that there were almost 1,000,000 patients with MI in the USA, with a 3-year mortality of nearly 25% [1]. Diabetes mellitus has been widely recognized as a major risk factor for cardiovascular disease, and carries the same risk of mortality as MI itself [2]. Previous studies have demonstrated that the prognosis of the coronary artery disease (CAD) in patients with DM is significantly worse than in patients with CAD alone [3]. However, the mechanisms that underlie this observation are not fully understood.

Mesenchymal stem cells (MSCs) transplantation has been widely used as an effective method of protecting the damaged myocardium and improving cardiac function [4]. MSCs can be readily isolated from bone marrow, adipose tissue, and umbilical cord blood. Ex vivo experiments indicate that MSCs express CD105, CD73, CD90, CD29 and CD166, but lack expression of CD45, CD34, CD14, CD11b, CD79α, CD19 and HLA-DR [5]. It has also been shown that MSCs transplantation improves heart function through various mechanisms, including angiogenesis, myogenesis, and inhibition of left ventricular (LV) remodeling [6]. The paracrine effects of MSCs have also been highlighted as an important mechanism responsible for improving cardiac function [7].

MSCs have been shown to secrete various cytokines, including vascular endothelial growth factor (VEGF), and hepatocyte growth factor (HGF), etc [8]. These cytokines play vital roles in paracrine function and contribute to cardiac repair through mechanisms involving cytoprotection, neovascularization and inhibition of apoptosis, all of which minimize ischemic reperfusion injury [9,10]. The beneficial effects of MSCs transplantation are well documented in both animal and clinical studies [11-13]. However, it is possible that age and other risk factors, such as the presence of DM might reduce the potential for MSCs differentiation and proliferation [14,15]. Age-related changes in MSCs function have previously been reported [16-18], but to date little is known about the effects of DM on MSCs proliferation.

Previous studies have described the biological characteristics of MSCs in a rat model [19] and in patients with CAD [13]. The present study was undertaken to investigate the differences in the gene expression profile of MSCs obtained from human subjects with CAD and DM, and with CAD alone, and to determine if this had the potential to alter the cardioprotective effect of hMSCs transplantation against MI in patients with CAD+DM.

## Methods

### Isolation and culture of human MSCs

Bone marrow collection for research purpose was approved by the Ethics Committee of the Shenyang Northern Hospital, Shenyang City, China. All patients provided informed consent and the study was conducted according to the ethical guidelines of the Declaration of Helsinki (1975).

MSCs were isolated and cultured as previously reported [13]. Briefly, 3 to 5 mL of bone marrow was aspirated from the sternum of patients with CAD who were undergoing coronary artery bypass graft surgery (CABG). Patients were divided into CAD+DM and CAD groups (n = 10 per group). The study inclusion and exclusion criteria are shown in Table 1, and patient demographic and clinical data are summarized in Table 2. The diagnosis of DM was based on The American Diabetes Association (ADA) diagnostic criteria [20].

**Table 1:** Inclusion and exclusion criteria

**Inclusion criterion**	**Male**
	Age between 50 and 60 years old
	Previous myocardial infarction with multiple vessels involved
	Type II diabetes mellitus for over 10 years
Exclusion criterion	Infectious, systemic immunologic diseases, malignancy, hepatic and nephritic dysfunction

**Table 2:** Patient demographic data

	**CAD+DM (n = 10)**	**DM (n = 10)**	**P-value**
Age, years	54.40 ± 3.10	55.8 ± 1.69	0.226
BMI, kg/m2	26.81 ± 2.74	25.97 ± 4.57	0.624
LVEF,%	43.9 ± 6.72	44.2 ± 4.89	0.910
Smoker, n	7	5	0.157
Hypertension, n	6	8	0.261
Hyperlipidemia, n	3	2	0.158
Target vessel, n			
LM	3	2	0.158
LAD	10	10	1.000
LCX	5	6	0.247
RCA	5	6	0.247
OM	5	4	0.247
Diagonal	4	3	0.572

Values are presented as n, or mean ± SD.

BMI body mass index; LVEF left ventricular ejection fraction; LM left main coronary artery; LAD left anterior descending coronary artery; RCA right coronary artery; OM obtuse marginal.

Bone marrow aspirates were placed in a 5 mL tube containing phosphate-buffered saline (PBS) and 1250U of heparin. The marrow samples were washed twice with PBS twice after centrifugation at 900 × g for 10 min to discard the fat layer. The residual cells were added into the equal volume of 1.073 g/mL Percoll solution in a 50 mL conical tube and centrifuged at 1100 × g/min for 30 min. The mononuclear cells were collected from the upper layer and interface, diluted with two volumes of PBS, and collected by centrifugation at 1100 × g/min. The cells were resuspended in low glucose Dulbecco’s Modified Eagle Medium (DMEM-LG; Gibco, Grand Isle, NY, USA) supplemented with 10% fetal bovine serum (Gibco, Mulgrave, Victoria, Australia), 100U/mL penicillin, and 100 mg/mL streptomycin. The nucleated cells were plated into 100 mm plastic culture dishes (Beckton Dickinson, San Jose, CA, USA) and incubated at 37°C in 5% CO_2_ and 95% humidity. The culture medium was replaced by new medium every 3 days. On each occasion, floating cells or non-adherent hematopoietic cells were removed. After 12 to 15 days of primary culture, the adherent cells were nearly 80% confluent. The cells were dissociated using 0.25% trypsin, replated at a ratio of 1:4 in 100 mm plastic culture dishes and grown to near confluence to expand the cells through successive passages.

Growth curves of MSCs from patients were depicted using the 3-(4, 5-dimethylthiazol-2-yl)-2, 5-diphenyltetrazlium bromide (MTT) assay. Briefly, cells from Passage 3 were cultured in 96-well plastic culture dishes at a density of 2.5 × 10^3^cells/well. After 1,2,3,4,5,6,7 and 8 days of culture, MTT (Sigma, St. Louis, MO, USA) dissolved in PBS was added to each well at a final concentration of 5 mg/mL, and the samples were incubated at 37°C for 4 h. Water-insoluble dark blue formazan crystals formed during MTT cleavage in actively metabolizing cells. These were dissolved in dimethyl sulfoxide (DMSO) (Gibco/Invitrogen, NY, USA). Optical density was measured at a wave length of 490 nm using a Bio-Rad 680 microplatereader (Bio-Rad, Califronia, USA)

### Phenotype analysis of hMSCs

The hMSCs from Passage 3 were trypsinized, incubated and stained with mouse anti-human antibody for 30 min at room temperature. The cells were then rinsed twice with PBS and resuspended in 500μL PBS after centrifugation at 900 × g. The cells were analyzed using a flow cytometer (Beckton Dickinson, San Jose, CA,USA). The antibodies used in this experiment were: CD34-PE, CD45-PE, CD29-PE and CD44-PE (Beckton Dickinson, San Jose, CA, USA).

Approximately 5 × 10^5^ cells per 100μL were labeled with primary mouse antibodies against human CD29, CD34, CD44 and CD45. Cells were incubated at 4°C for 30 min and washed. Mouse IgG1-PE (Beckton Dickinson, San Jose, CA, USA) was used as an isotype control [21].

### Gene expression profiling and protein validation

The hMSCs from Passage 3 were used for RNA extraction. Total RNA (2 μg) from human CAD-MSCs and CAD+DM-MSCs (n = 3 per group) were used for microarray analysis. Affymetrix (Santa Clara, USA) GeneChipHuman Genome U133 Plus 2.0 Arrays, which allowed analysis of 47,000 transcripts, were performed in triplicate and analyzed with Affymetrix Microarray Suite (MAS 5.0). The transcripts were annotated using various databases to compile a list of apoptosis candidates. All the experiments of gene expression profiling were authorized to be carried out by CapitalBio Corporation (Beijing, China).

Gene expression profiling was determined by Western blot analysis for proteins expressed by selected genes. Briefly, the hMSCs were treated with cell lysis buffer (Promega, Madison, WI, USA). Concentration was determined using a BCA protein assay (Pierce, Rockford, IL, USA). Total proteins (20 μg) were separated using 12.5% sodium dodecyl sulfate-polyacrylamide gel electrophoresis and transferred to a 0.2 mm nitrocellulose membrane. The membrane was blocked in PBS buffer containing 0.2% Tween 20 and 5% non-fat milk for 1 h. The membrane was then incubated overnight at 4°C with rabbit Bcl-2 protein polyclonal antibody (1:2000dilution; Abcam, Cambridge, MA, USA). Housekeeping protein β-actin was employed as loading control. Antibody binding was detected using horseradish peroxidase conjugated secondary antibody, and visualized by an ECL kit (Amersham Biosciences, Piscataway, NJ, USA).

### Myocardial infarction formation and hMSCs transplantation

Male Sprague-Dawley (SD) rats weighing 280 to 300 g were divided into three groups (CAD+DM group; CAD group; control group, n = 14 per group). All animals received humane care, and all animal protocols complied with the institution’s guidelines.

Rats were anesthetized by intraperitoneal injection with pentobarital (50 mg/kg), intubated via an endotracheal cannula and mechanically ventilated. A left lateral thoracotomy was performed. The proximal portion of the left anterior descending artery was ligated with a 6-0 Prolene (Ethicon, Somerville, NJ, USA) suture. MSCs from Passage 3 were used for transplantation. The MSCs were dissociated from the culture dishes with 0.25% trypsin, neutralized with culture medium, washed with PBS and collected by centrifugation at 900 × g for 5 min at room temperature. The cells were then suspended in culture medium at a concentration of 2 × 10^6^ cells in 50μL and were kept on ice until transplantation. Animals in the CAD+DM (n = 11) and CAD (n = 9) groups received a sub-epicardial injection of hMSCs obtained from patients with CAD+DM and CAD respectively. The cells were injected into the infarcted scar and adjacent myocardium. The control group (n = 11) received injections of culture medium into the same area. Intramuscular penicillin G benzathine (100,000U/kg) was used to prevent infection. The hMSCs from different patients were injected respectively. Immunosupprerssion was provided by daily intramuscular administration of cyclosporine (10 mg/kg/day; Novartis, Switzerland) for 4 weeks post transplantation.

### Echocardiography

A blinded investigator performed transthoracic echocardiographic studies on the anesthetized rats. Left ventricle dimension and function were assessed immediately prior to myocardial infarction, and at 1 and 4 weeks after hMSCs transplantation. Images were recorded using a 12-MHz high frequency liner phased-array transducer (Philips SONOS 5500, Bothell, WA, USA). Left ventricular end diastolic and systolic dimensions were derived from two-dimensionally targeted M-mode tracings obtained along the para-sternal short-axis view of the left ventricle at the papillary muscle level. Ejection fraction (EF) and fractional shortening (FS) were calculated. All measurements were performed and averaged over three consecutive cardiac cycles.

### TUNEL (Terminal Doxynucleotidyl Transferase-mediated dUTP-x Nick End Labeling) staining

The histochemical detection of apoptotic cells was performed as previously reported [22]. The tissue blocks were fixed in 4% paraformaldehyde and incubated with proteinase K. Fragments of DNA in the tissue sections were analyzed using a TUNEL detection kit (Promega Corporation, Madison, WI, USA). For each slide, color images of 10 separate fields were captured randomly and digitized. Cells with clear nuclear labeling were defined as TUNEL-positive cells. The apoptotic index was calculated as the number of TUNEL-positive cells/ total number of myocytes.

### Immunohistochemistry and Western-blot analysis

Myocardial tissue was embedded in paraffin and cut into 5 μm sections. Detection of Bcl-2 expression was performed as described previously [23]. Tissue sections were exposed overnight to rabbit Bcl-2 protein polyclonal antibody (1:2000 dilution; Abcam, Cambridge, MA, USA) at 4°C, washed in PBS and incubated with biotinylated goat anti-rabbit IgG for 60 min at 37°C. After two washing steps, sections were exposed to streptavidin-horseradish-peroxidase complex for 30 min at 37ºCand visualized with 3, 3’-diaminobenzidine, embedded in glycerol gelatin. Images were captured digitally and analyzed using IPP version 6.0. Cytoplasmic staining was considered positive, and scored as: absent (-); weakly positive (+), moderately positive (++) or strongly positive (+++). Myocardial tissue samples were homogenized in RIPA buffer and the protein expression of Bcl-2 and VEGF were identified by Western blotting as described above.

### Statistical analysis

Data were analyzed using SPSS version 12.0 for Windows (SPSS, Chicago, IL, USA). All variables were presented as means and standard deviations (±SD). The *t*-test was used to compare treatments in the in vitro experiments. The results of hMSCs transplantation into rat models were tested using one-way analysis of variance. Tukey’s method was used for post-hoc analysis. Values of P < 0.05 were considered statistically significant.

## Results

### Growth characteristics of hMSCs

**Figure 1:**
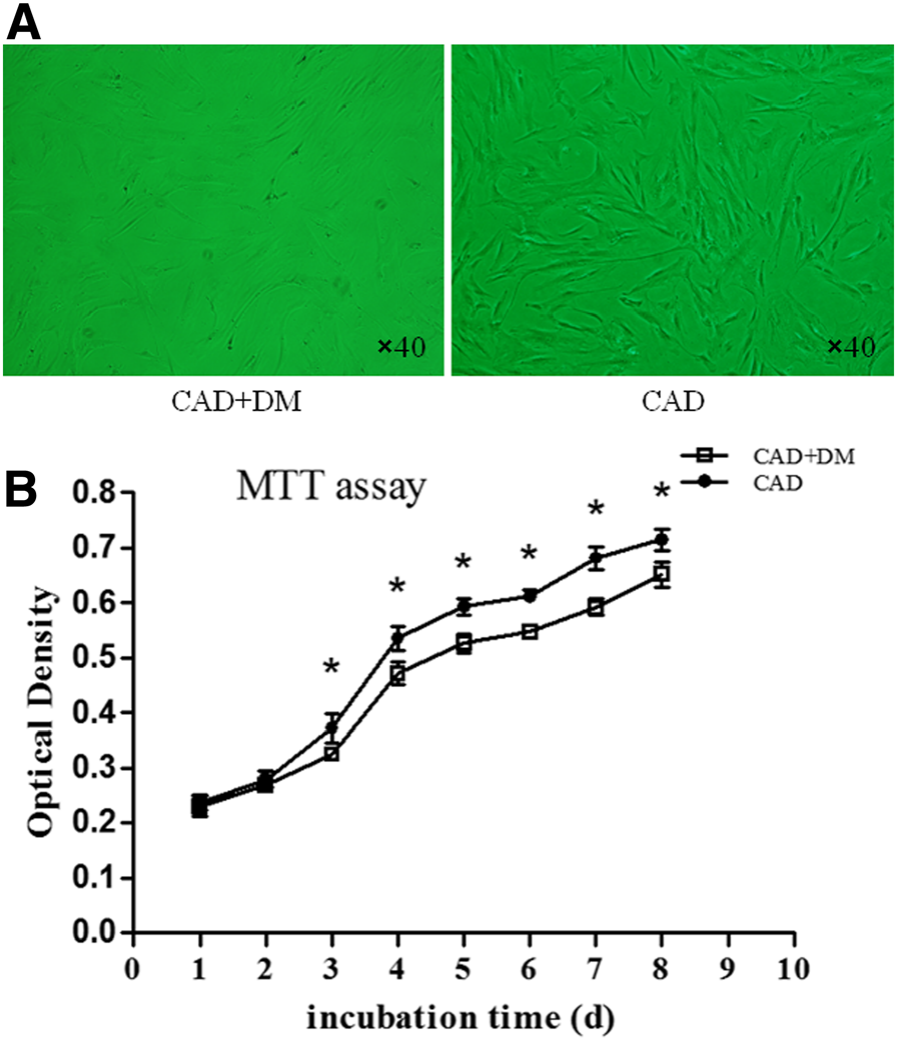
**Characteristic of hMSCs in Passage-3 and proliferative abilities of hMSCs between each group. **(**A**) In Passage 3, the hMSCs from CAD+DM group showed more flattened appearance and a larger size than those from the CAD group. (**B**) A MTT assay was performed and the optical densities of the two groups were detected at 490 nm. The proliferative potential of MSCs from the CAD+DM group decreased significantly compared with CAD group at each time point of the log phase and plateau phase after 2 days post transplantation (*P < 0.05 vs. CAD group).

The hMSCs were tightly attached to the culture dishes after 24 h. They appeared as spindle shaped cells after 3 to 4 days’ culture of the primary passage, after which they proliferated rapidly. After 12 to 15 days of primary culture, the hMSCs reached nearly 80% confluences. The hMSCs in passage 3 from the CAD+DM group had a more flattened appearance were larger in size than those from the CAD group (Figure 1A).

### Proliferative abilities of MSCs from each group

Growth curves were characterized by an initial lag phase (during the first 2 days) followed by a log phase (from 3 to 7 day) during which cells divided at exponential rates. This was followed by a plateau phase after Day 8. The proliferative potential of hMSCs obtained from patients with CAD+DM was significantly impaired relative to that seen in cells from patients with CAD (P <0.05). These differences were apparent at each time point after Day 2 (Figure 1B).

### Phenotype of human MSCs

**Figure 2:**
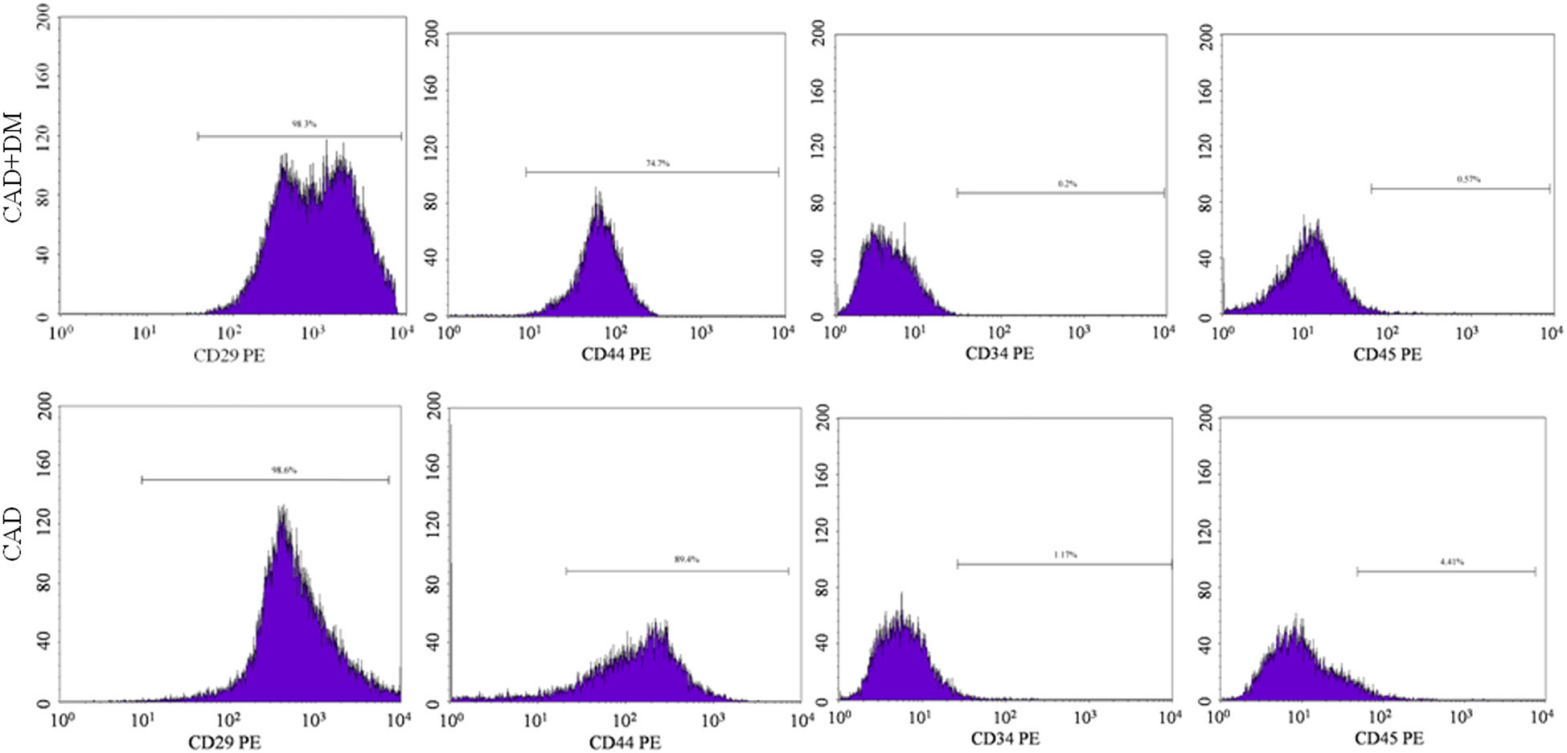
**The phenotypic nature of human MSCs.** The phenotype of human MSCs from CAD+DM and CAD groups were positive for CD29, CD44 and negative for CD34, CD45.

Human MSCs contained a unique phenotypic population which was identified by flow cytometric analysis of expressed surface antigens. All hMSCs were uniformly positive for CD29, CD44 and negative for CD34 and CD45 (Figure 2).

### Differences of genes expression profile of hMSCs between CAD+DM and CAD groups

**Figure 3:**
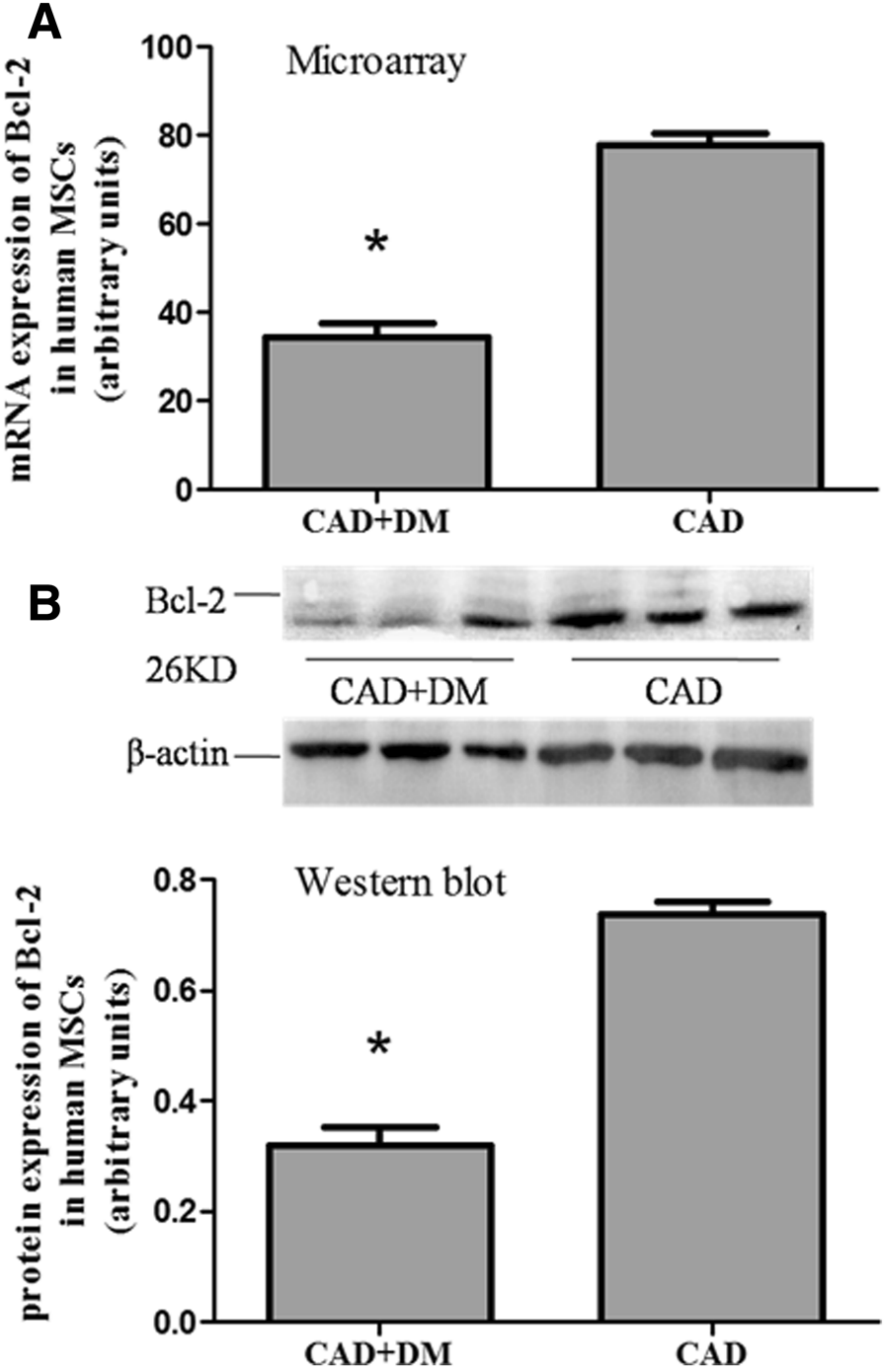
**Bcl-2 expression in human MSCs. **(**A**) Levels of Bcl-2 expression estimated by Affymetrix microarray analysis show a nearly 2.5-fold down-regulation in CAD+DM-MSCs compared with CAD-MSCs. (**B**) Western blot validation of protein levels demonstrated a great than 2-fold down-regulation in CAD+DM-MSCs compared with CAD-MSCs. (*P < 0.05 vs. CAD group).

Three transcripts were dramatically up-regulated (TNFRSF10B, TNFRSF21, NGF) and six transcripts that were down-regulated (EPR1, BIRC5, HELLS, BCL2, TNFRSF1B, CASP1) in the CAD+DM group relative to the CAD group (Table 3). In addition, expression of Bcl-2 mRNA was significantly lower in the CAD+DM group than in the CAD group (Figure 3A).

**Table 3:** Differential apoptosis-related genes from hMSCs obtained from CAD+DM and CAD

**ProbeSet ID**	**Gene title**	**Gene symbol**	**CAD+DM/CAD**
206814_at	Nerve growth factor(beta polypeptide)	NGF	4.1658
209295_at	Tumor necrosis factor receptor superfamily, member 10b	TNFRSF10B	2.3979
218856_at	Tumor necrosis factor receptor superfamily, member 21	TNFRSF21	2.2182
1555826_at	Effector cell peptidase receptor 1 (non-protein coding)	EPR1	0.2846
202094_at	Baculoviral IAP repeat-containing 5	BIRC5	0.1978
203508_at	Tumor necrosis factor receptor superfamily, member 1B	TNFRSF1B	0.3262
203685_at	B-cell CLL/lymphoma 2	BCL2	0.4131
209970_x_at	Caspase 1, apoptosis-related cysteine peptidase (interleukin 1, beta, convertase)	CASP1	0.3542
220085_at	Helicase, lymphoid-specific	HELLS	0.4298

To confirm the gene expression profile results, Bcl-2 protein was evaluated in vitro and Western bolt analysis was performed on cell samples at the same time points. These results also showed that the expression of Bcl-2 protein was significantly lower in the CAD+DM group than in the CAD group (Figure 3B).

### Evaluation of myocardial function

Transplantation of hMSCs into the infarcted border of zone of rats with experimentally induced CAD significantly improved left ventricular function. There were no remarkable differences in EF and FS between the three groups preinfarction or at 1 week postinfarction.

In the CAD+DM and CAD groups, both EF and FS increased significantly, relative to the control group, after MSC transplantation. However, EF and FS were significantly lower in the CAD+DM group than in the CAD group.

**Figure 4:**
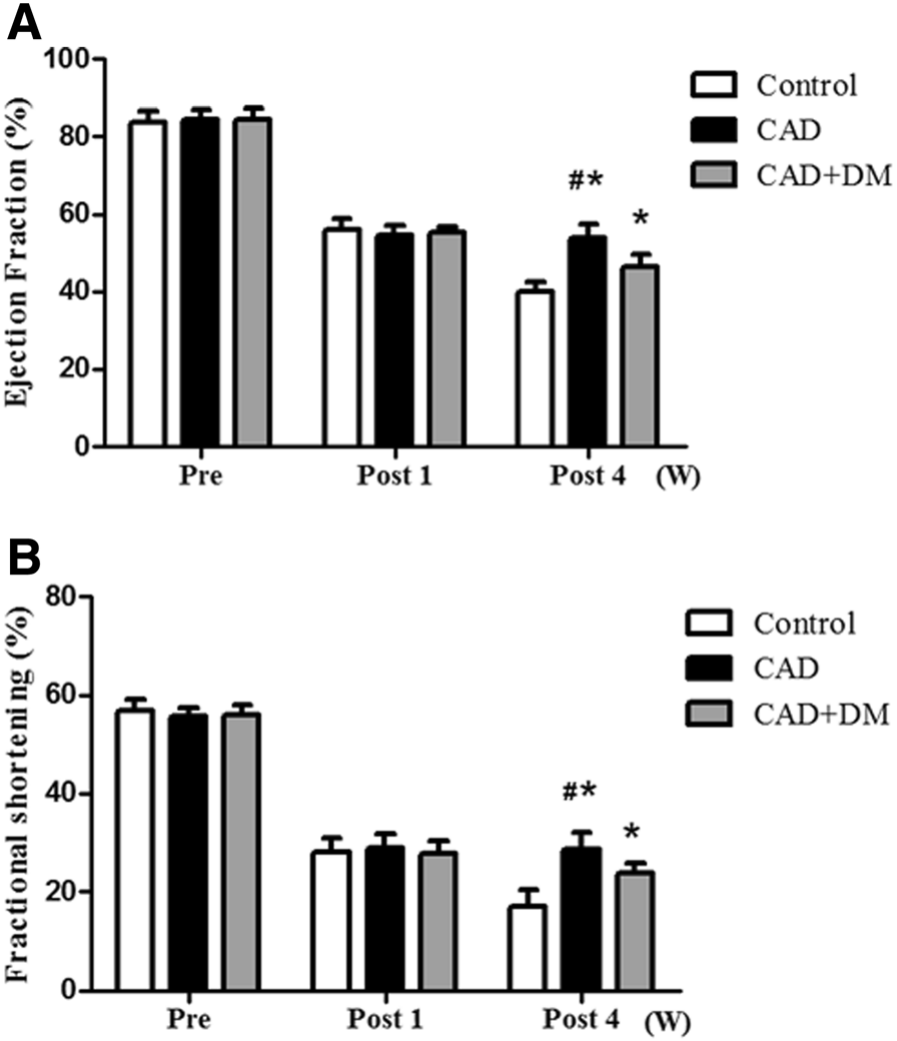
**Evaluation of myocardial function.** Ejection fraction EF (**A**) and fractional shortening FS (**B**) were not significantly changed in any of the three groups at 1 week post infarction. However, EF and FS increased significantly in CAD+DM and CAD groups after MSC transplantation. EF and FS were significantly lower in the CAD+DM group than in the CAD group after 4 weeks. (*P < 0.05 CAD+DM and CAD groups compared with control group, #P < 0.05 CAD+DM group compared with CAD group).

Contractile function was impaired in all three groups after infarction, but significantly improved after 4 weeks in animals transplanted with hMSCs (CAD+DM and CAD groups) relative to values in the control group. Improvements in contractile function were significantly less marked in the CAD+DM group than in the CAD group (Figure 4).

### Myocardial apoptosis after hMSCs transplantation

**Figure 5:**
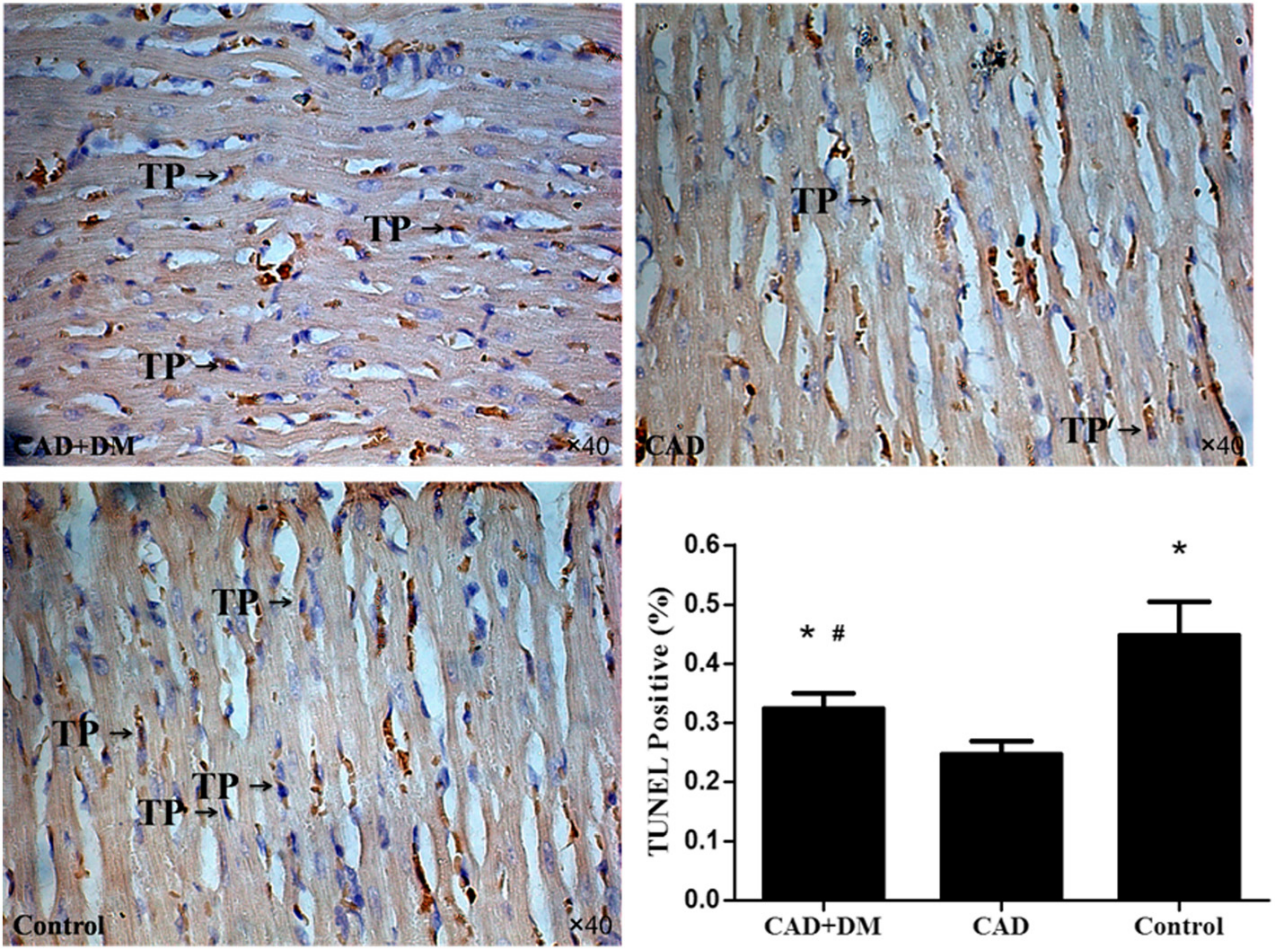
**Myocardial apoptosis 4 weeks after cells transplantation.** Myocardial apoptosis was detected by TUNEL staining. TUNEL-positive cells (TP indicated) decreased significantly in CAD+DM and CAD groups compared with control group. However, the number of TUNEL-positive cells was significantly higher in the CAD+DM group than in the CAD group. (*p < 0.05 vs. control group, ^#^P < 0.05 vs. CAD group).

The degree of apoptosis 4 week after hMSCs transplantation was significantly lower in the CAD+DM and CAD groups than in the control group. However, myocardial apoptosis was significantly higher in the CAD+DM group than in the CAD group (Figure 5).

### Protein expression in infarcted myocardium

**Figure 6:**
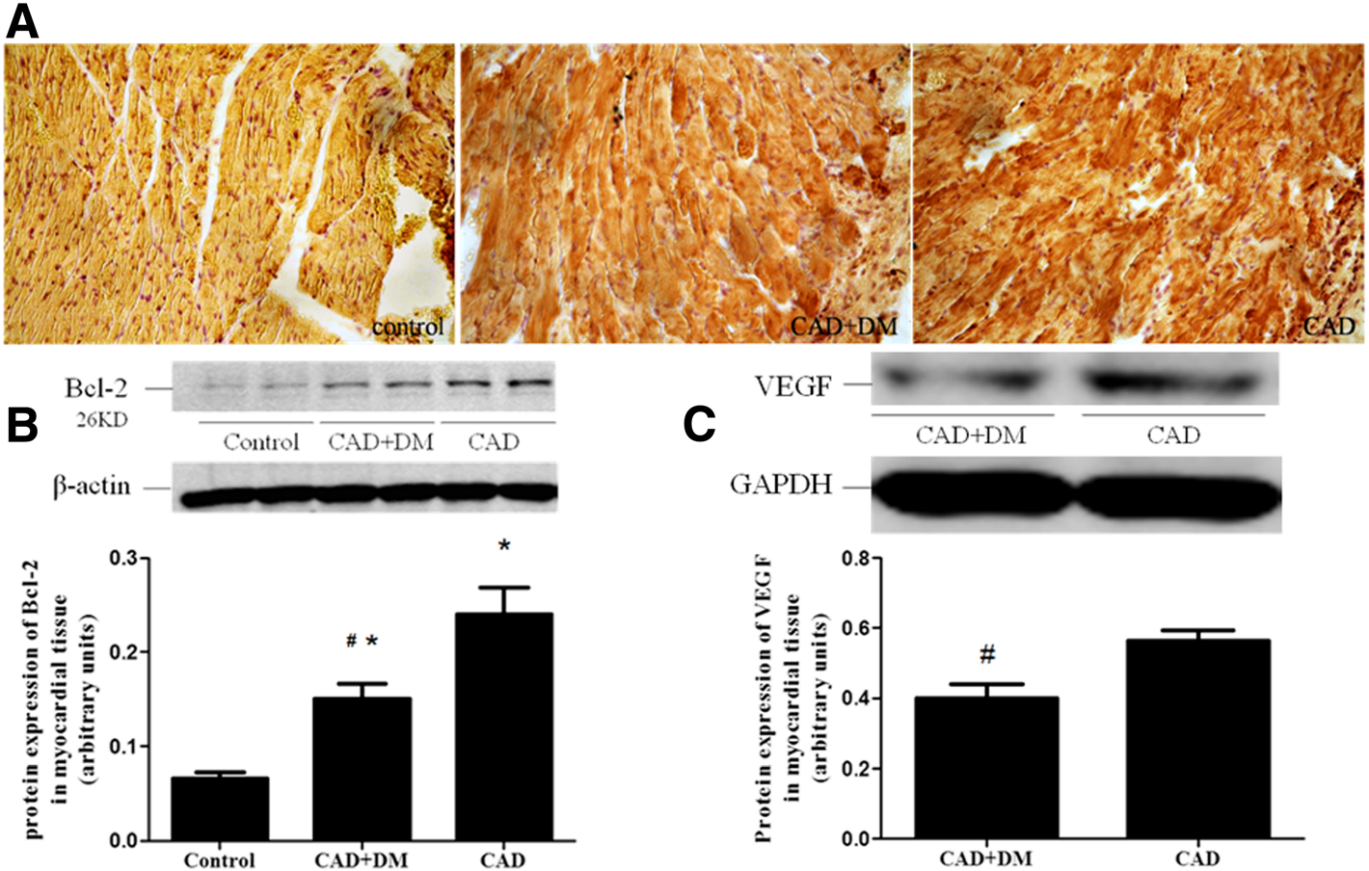
**Protein expression in infarcted myocardium. **(**A**) Immunohistochemical detection of Bcl-2 expression in infarcted myocardium from control (+), CAD+DM (++) and CAD (+++) groups. (**B**) Western-blot analysis shows that hMSCs transplantation from patients with CAD+DM and CAD significantly increased Bcl-2 expression in the myocardium compared with the control group. However, Bcl-2 protein level was significantly lower in the CAD+DM group than in the CAD group. (**C**) Western-blot analysis shows that VEGF expression after hMSCs transplantation in CAD+DM group was significantly lower than in the CAD group. (*P < 0.05 vs. control group, ^#^P < 0.05 vs. CAD group).

Protein expression of Bcl-2 following hMSC transplantation in the CAD+DM and CAD groups were both significantly higher than in the control group, as evidenced by immunohistochemical staining and Western-blot analysis. However, Bcl-2 levels in the CAD+DM were significantly lower than in the CAD group. Meanwhile, VEGF levels in the CAD+DM were also significantly lower than in the CAD group (Figure 6).

## Discussion

The present study demonstrated that hMSCs, isolated from the sternum had a unique appearance and phenotype similar to the MSCs isolated from iliac bone marrow. A previous study demonstrated that hMSCs from Passage 1 to 5 had significantly greater proliferative potential than those from later passages [13]. We therefore chose hMSCs from Passage 3 for all our *in vitro* and *in vivo* experiments.

We demonstrated there was a significant difference in proliferation and gene expression profiling of hMSCs derived from patients with CAD+DM relative to those derived from patients with CAD only. These findings provided initial evidence that DM reduces the proliferation of hMSCs *in vitro*. The current results were consistent with previous reports in which they found that endothelial progenitor cells were depleted even in DM patients without clinical evidence of macrovascular disease [24]. We also showed that Bcl-2 as well as other differential genes may play a crucial role in hMSC proliferation. The other important finding was that transplantation of hMSCs from CAD patients into rats with experimentally induced myocardial infarction improved cardiac contractility and attenuated apoptosis of cardiomyocytes. These effects were also weakened in MSCs derived from patients with CAD+DM, possibly due in part to reduced expression of Bcl-2 in these cells. These results were consistent with those from a previous study using a rat model [19].

In our study, we identified several differentially expressed genes related to apoptosis. From differential genes, TNFRSF10B, TNFRSF21 and TNFRSF1B are a member of the TNF-receptor superfamily, which deliver signals for cell death, survival, proliferation and differentiation. However, their effects on apoptosis are diverse. TNFRSF10B, TNFRSF21 can be activated by tumor necrosis factor-related apoptosis inducing ligand and transducer apoptosis signals. In contrast, TNFRSF1B plays a vital role in preventing apoptosis [25]. Moreover, BIRC5, which positively correlated with the expression of Bcl-2, is a member of the inhibitor of apoptosis gene family and take part in the prevention of apoptotic cell death [26]. From all the differential genes related to apoptosis, Bcl-2 was selected for further study since our previous studies demonstrated that Bcl-xl gene transfer has a cardioprotective function against ischemia/reperfusion injury [27,28]. Both Bcl-2 and Bcl-xl belong to the Bcl-2 family, and are overexpressed in B-cell lymphoma [29]. Bcl-2 family of proteins acts as critical regulators of pathways involved in anti-apoptosis and inhibition of cell death [30]. It has also been shown that Bcl-2 contributes to cardiac protection during ischemic conditions, where it acts as one of the regulators of the metabolic functions of mitochondria [31]. In the present study, mRNA and protein expression of Bcl-2 were significantly lower in the CAD+DM group than in the CAD group, suggesting that Bcl-2 expression in patients with CAD might be impaired by DM.

MSCs exhibit the property of immune-tolerance whereby they express low levels of major histocompatabilty complex (MHC) and co-stimulant molecules [32]. This means that MSCs are generally safe and effective when used for allo-transplantion [11]. However, post-transplant rejection has previously been reported in a xenogenic model [33], and for this reason we used, cyclosporine to suppress the immune response in our study.

We also showed that hMSCs transplantation improved myocardial but that the improvement was significantly more marked with cells derived from patients with CAD than with those derived from patients with CAD+DM. The findings that MSCs transplantation improves heart function after myocardial infarction and that DM may weaken myocardial protective function of hMSCs transplantation significantly are in accordance with previous studies [13,34]. In our study, myocardial tissue from the infarcted zone and border was determined by TUNEL, imunohistochemistry and Western-blot analysis. We found that apoptosis of myocardial cells increased dramatically in CAD+DM group compared with CAD group, in accordance with a previous study in a rat model [19]. We also showed that, expression of Bcl-2 decreased markedly in the CAD+DM group compared with CAD group. Previous studies revealed a reduced expression of VEGF in the myocardium in diabetes [35]. However, there is no difference between the two groups at mRNA level in Gene Chip results of cultured hMSCs. On the contrary, protein expression of VEGF after hMSCs transplantation decreased significantly in the CAD+DM group compared with CAD group. This might be due to the elevated VEGF secretion induced by high-level expression of Bcl-2 in response to hypoxic condition [36]. Thus, different levels between protein and mRNA expression existed in cell culture under normoxic condition and infarcted myocardium under hypoxic condition. Thus, inhibition of Bcl-2 expression may result in increased apoptosis which in turn decreases myocardial protection; decreased secretion of VEGF may one of the possible reasons. It has been shown that the function of MSCs can be modulated by introduction of specific genes. [36]. It is therefore possible that hMSCs from CAD+DM patients modified with Bcl-2 gene may have improved expression levels of Bcl-2 protein and enhanced ability to protect the ischemic myocardium. At the same time, the method of human stem cell based three dimensional microtissues may also represent a translational therapy strategy which may enhance cellular retention, survival and integration [37]. Both of above can be studied in deep-going way in the future.

### Limitations

Previous studies have demonstrated that aging impairs the quantity, quality and mobilization capacity of MSCs [14,38]. Thus, hMSCs were obtained from patients 50 to 60 years of age, which limited the number of relative younger patients with CAD+DM. This may have compromised the capacity of MSCs to offer myocardial protection. The relatively small sample size in our study may also have impacted our results to some extent. Our findings therefore need to be substantiated in larger populations of patients of different age groups.

## Conclusions

The present study indicates that hMSCs from patients with CAD+DM and CAD have proliferative properties, and that transplantation of hMSCs from all patients improved heart function in rats with experimentally-induced myocardial infarction. The ability to proliferate and preserve myocardial function decreased significantly in MSCs obtained from patients with CAD+DM compared with those obtained from patients with CAD. The differential effects of transplantation of different human MSCs might result from differences of Bcl-2 protein expression, which determine the extent of anti-apoptosis.

## Abbreviations

MI: Myocardial infarction;DM: Diabetes mellitus;CAD: Coronary artery disease;MSCs: Mesencymal stem cells;LV: Left ventricular;VEGF: Vascular endothelial growth factor;HGF: Hepatocyte growth factor;hMSCs: human MSCs;CABG: Coronary artery bypass graft surgery;ADA: American diabetes association;PBS: Phosphate-buffered saline;DMEM-LG: Low glucose Dulbecco’s modified Eagle Medium;MTT: 3-(4,5-dimethylthiazol-2-yl)-2, 5-diphenyltetrazlium bromide;DMSO: Dimethyl sulfoxide;EF: Ejection fraction;FS: Fractional shortening;TUNEL: Terminal doxynucleotidyl transferase-mediated dUTP-x Nick End Labeling

## Competing interests

The authors declare that they have no competing interests.

## Authors’ contributions

HW and JH made substantial contributions to the conception and design of this study. XX participated in designing the study, performing the experiments. HJ participated in acquisition of hMSCs. YL carried out the cells culture studies, acquisition of data, performed the statistical analysis and drafted the manuscript. TL carried out animal experiments and performed Immunohistochemistry analysis. ZL carried out TUNEL staining and Western-blot analysis. All authors read and approved the final manuscript.
